# Real-world eligibility for platinum doublet plus immune checkpoint inhibitors in extensive-stage small-cell lung cancer

**DOI:** 10.3389/fonc.2022.1002385

**Published:** 2022-09-15

**Authors:** Rebekah Rittberg, Bonnie Leung, Zamzam Al-Hashami, Cheryl Ho

**Affiliations:** ^1^ Department of Medical Oncology, BC Cancer, Vancouver, BC, Canada; ^2^ Sultan Qaboos Comprehensive Cancer Care and Research Center, Muscat, Oman

**Keywords:** small-cell lung cancer (SCLC), real world, durvalumab, atezolizumab, platinum doublet, immunotherapy

## Abstract

**Introduction:**

Small cell lung cancer (SCLC) is a rapidly progressing aggressive malignancy. Durvalumab in CASPIAN and atezolizumab in IMPower133 were found to improve overall survival (OS) for extensive-stage SCLC. Here we evaluate the proportion of real-world ES SCLC patients who may be eligible for first-line immune checkpoint inhibitor (ICI) with platinum doublet.

**Methods:**

A retrospective cohort analysis was conducted of referred ES SCLC between 2015 and 2017 in British Columbia, Canada. Patient demographics, staging, treatment, and survival data were collected through the Cancer Registry. Retrospective chart review was completed to extract past medical history and missing variables. CASPIAN/IMPower133 excluded patients with autoimmune diseases, active infection, and performance status (PS) ≥2.

**Results:**

Between 2015 and 2017, 349 patients were diagnosed with ES SCLC. In patients who received platinum-doublet chemotherapy (n=227), 15 had medical contraindication to ICI: inflammatory bowel disease (n=4), rheumatoid arthritis (n=4), idiopathic pulmonary fibrosis (n=3), lupus (n=1), Sjogren’s (n=1), Takayasu arteritis (n=1), and active tuberculosis (n=1). ECOG PS was 0–1 in 96 (45%), PS was 2 in 61 (29%), and ≥3 in 51 (10%). Prior to cycle 1, 82 (36%) patients were eligible for ICI in addition to platinum doublet, 23% of the entire ES population. After cycles 1 and 2, additional 15 (7%) and 8 (4%) patients became PS 0–1, respectively. mOS for ES SCLC who received first-line platinum doublet, non-platinum chemotherapy, and best supportive care was 8.4 1.9 and 1.5 months (p<0.001).

**Discussion:**

By CASPIAN/IMpower133 trial eligibility, only 36% of our real-world platinum-treated patients would have been eligible for the addition of ICI, which is 23% of the entire ES population in one Canadian province. After one or two cycles of chemotherapy, an additional 11% of patients showed PS improvement to 0–1. While the results of CASPIAN/IMpower133 are practice-changing, the majority of the patients will not meet clinical trial eligibility and clinical trials including patients with poor PS are necessary.

## Introduction

Lung cancer continues to be the global leading cause of cancer-related death (1). Although non-small-cell lung cancer (NSCLC) accounts for most lung cancer diagnoses, there have been significant treatment advancements over the last 10 years including the maintenance pemetrexed, identification and targeting of driver mutations, and use of immune checkpoint inhibitors (ICIs) each resulting in improved survival in treated patients (2–5). Unfortunately, similar advances in small-cell lung cancer (SCLC) have not been observed with first-line treatment remaining platinum etoposide for over two decades.

SCLC accounts for just 13% of new lung cancer diagnosis and is characterized as a rapidly progressive neuroendocrine tumor with two-thirds of patients diagnosed with extensive-stage (ES) disease (6). Smoking continues to be the primary risk factor for SCLC with >98% of new SCLC cases having a smoking history (7). ICIs were first evaluated in SCLC post platinum-based chemotherapy. Pembrolizumab was approved as ≥2 lines of therapy based on an overall response rate of 19.3% by the Food and Drug Administration (FDA) (8). Results from second-line nivolumab were originally encouraging and received FDA-accelerated approval; however, confirmatory trials did not find an improved overall survival (OS) and thus the indication was withdrawn (9, 10).

First-line treatment has been unchanged for multiple decades given the high responsiveness to first-line platinum-based chemotherapy. Current SCLC treatment is not guided by molecular profiling due to the lack of targetable mutations (11). This has led to the evaluation of ICI in the first-line setting in conjunction with platinum-based chemotherapy in multiple trials. CASPIAN evaluated platinum-based chemotherapy alone and platinum-based chemotherapy in addition to durvalumab with and without tremelimumab. Platinum-based chemotherapy plus durvalumab improved OS compared with platinum-based chemotherapy alone from 10.3 to 13.0 months; however, the addition of durvalumab and tremelimumab did not improve OS (12, 13). IMpower133 similarly considered atezolizumab in addition to platinum-based chemotherapy with an improved OS from 10.3 to 12.3 months (14). KEYNOTE-604 evaluated platinum-based chemotherapy with or without pembrolizumab, which found a non-significant trend toward improved OS (15). However, in the management of real-world SCLC patients, contraindications limit the use of ICIs, most notably history of autoimmune diseases, active infection, and poor performance status (PS).

SCLC patients continued to have poor outcomes and unmet systemic therapy needs. ICIs, in addition to platinum-doublet chemotherapy, are the new first-line standard of care in ES or relapsed SCLC; however, it is not known what proportion of ES SCLC patients will be eligible to benefit from combination therapy. Here we retrospectively evaluate the eligibility of first-line ICIs in a pre-ICI population to forecast the expected use of ICIs in a Canadian landscape, which currently does not publicly fund ICIs in addition to platinum doublet.

## Methods

### Population

British Columbia has a population of 5.1 million people with centralized cancer-care delivery through six cancer centers and over 40 satellite community oncology network sites. The Outcomes and Surveillance Integration System contains diagnosis information, baseline characteristics, and patient outcomes for all referred lung cancer patients in British Columbia. Currently, in Canada, ICIs in addition to platinum doublet are not reimbursed through the public medical system, and durvalumab is only available with the addition of chemotherapy through a patient access program.

A retrospective cohort study was conducted at BC Cancer of patients diagnosed with SCLC between 1 January 2015 and 31 December 2017. Baseline patient demographics, Eastern Cooperative Oncology Group (ECOG) PS, cancer staging, treatment (chemotherapy and radiotherapy), and survival were collected using the Outcomes and Surveillance Integration System, electronic medical records, and billing administration database for chemotherapy. Past medical history and missing data were obtained through a manual chart review.

### Statistical analysis

Statistical Package for the Social Sciences software version 28 was used to produce descriptive statistics using chi-square and Mann–Whitney U tests. OS was calculated from date of diagnosis using the Kaplan–Meier curves and compared using the log rank test. Patients were censored at last known follow-up. Statistically significant p-value were set at <0.05.

### Ethics statement

This study was conducted with the University of British Columbia/BC Cancer Research Ethics Board approval (H19-02381). A waiver of consent was granted to extract and analyze data for this retrospective review.

## Results

Between 2015 and 2017, 519 patients were diagnosed with SCLC of which 349 (67%) were diagnosed with ES SCLC. The baseline characteristics are found in **Table 1**. Within the population, 2% of the patients were lifelong non-smokers with 62% actively smoking at the time of cancer diagnosis. Baseline PS was 0–1 for 114 (33%) patients, PS 2 for 90 (26%) patients, and ≥3 for 139 (40%) patients of the population.

**Table 1:** Baseline characteristics and treatment history of extensive stage small cell lung cancer patients.

N (%)	Extensive stage (n = 349)
Age (median), years	68
Sex	
Male Female	173 (49%) 176 (51%)
Smoking Status	
Never Former Active Unknown	6 (2%) 124 (35%) 215 (62%) 4 (1%)
Smoking years (median)	50
ECOG PS	
0-1 2 3-4 Unknown	114 (33%) 90 (26%) 139 (40%) 6 (2%)
CNS Metastases	118 (34%)
First line chemotherapy	
Platinum doublet Cisplatin doublet Carboplatin doublet Switch platinum doublet Single agent etoposide Other	227 (65%) 76 (34%) 125 (55%) 26 (11%) 24 (7%) 2 (1%)
Second line chemotherapy Platinum doublet Single agent etoposide Topotecan Irinotecan CAV	66 (19%) 28 (8%) 4 (1%) 13 (4%) 11 (3%) 10 (3%)
Third line chemotherapy Platinum doublet Single agent etoposide Topotecan Irinotecan CAV	15 (4%) 3 (1%) 0 5 (1%) 4 (1%) 1 (<1%)
Thoracic Radiation	130 (37%)
PCI	13 (4%)
WBRT	117 (34%)

N, number; ECOG PS, Eastern Cooperative Oncology Group Performance Status, CNS, central nervous system; PCI, prophylactic cranial radiation; WBRT, whole brain radiation.

**Figure 1:**
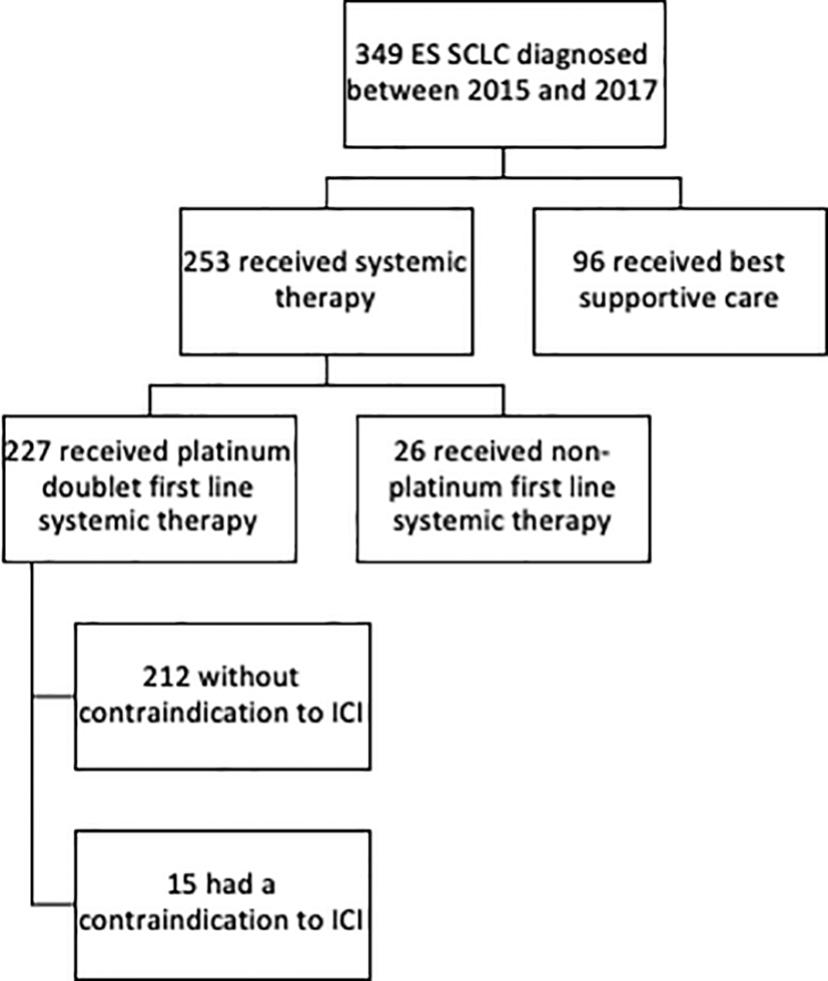
Consort diagram of extensive stage small cell lung cancer dataset assessing eligibility for ICI.

Systemic therapy was administered to 253 (72%) patients with 227 (90%) patients receiving first-line platinum-doublet chemotherapy (**Table 1** and **Figure 1**). Of the ES patients who received first-line platinum doublet, 34% received cisplatin, 55% carboplatin, and 11% switched between cisplatin and carboplatin. Only one cycle of platinum doublet was received by 37 patients, and 10 patients received only two cycles. First-line single-agent etoposide was administered to 24 (9%) patients, and the remaining 96 (28%) received best supportive care alone. Two lines of therapy were received by 155 (44%) patients and ≥3 lines by 4%.

Medical contraindications to ICIs were found in 15 patients who received platinum-doublet chemotherapy. These included inflammatory bowel disease (n=4), rheumatoid arthritis (n=4), idiopathic pulmonary fibrosis (n=3), lupus (n=1), Sjogren’s (n=1), Takayasu arteritis (n=1), and active tuberculosis (n=1). There were additional five patients who had autoimmune diseases, namely mild psoriasis (n=3) and stable hyperthyroidism (n=2) that were considered ICI eligible.

**Figure 2:**
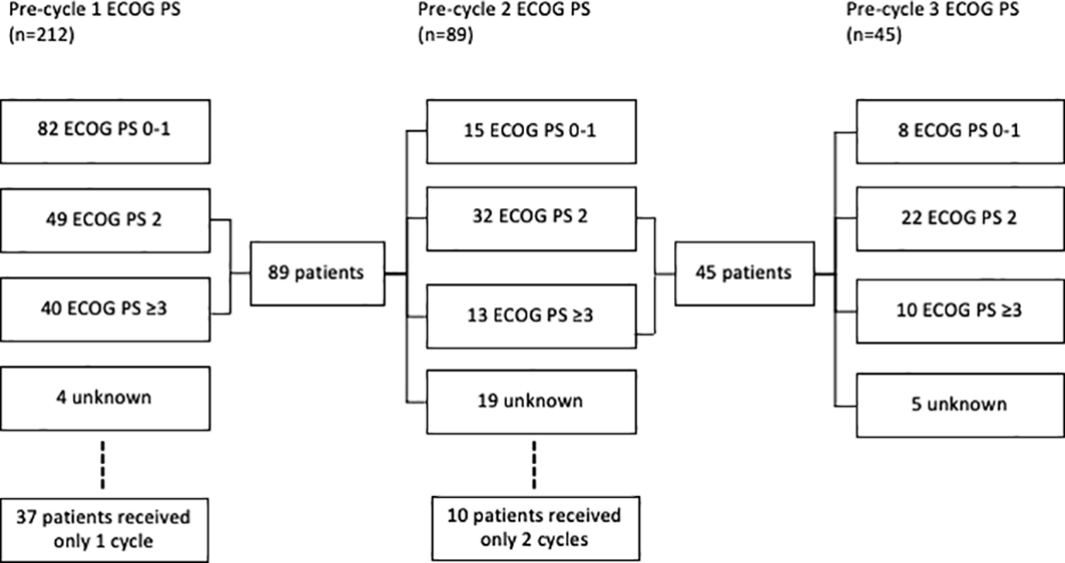
Consort diagram of ECOG of small cell lung cancer patients treated with platinum doublet prior to cycle 1, 2, and 3.

In patients who received platinum-doublet chemotherapy, the baseline ECOG PS was 0–1 in 96 (45%), 2 in 61 (29%), and ≥3 in 51 (10%) (**Figure 2**). Prior to cycle 2, 15 (7%) patients with ECOG PS ≥2 improved to PS 1. Prior to cycle 3, eight (4%) patients that were PS ≥2 prior to cycle 2 improved to PS 1.

Prior to cycle 1, 96 (45%) patients were eligible for ICIs in addition to platinum doublet if inclusion criteria included ECOG PS 0–1, 28% of the entire ES population. Baseline characteristics were similar between eligible and ineligible patients (**Table 2**). If eligibility was extended to include ECOG PS 2, an additional 61 (29%) patients would have become eligible. With the inclusion of PS 0–1, after cycle 1 was administered, another 15 (7%) patients would have become eligible, and after cycle 2, an additional 8 (4%) patients would have been eligible.

**Table 2:** Extensive stage small cell lung cancer patients who received first line platinum doublet by eligibility for first line chemotherapy with immune checkpoint inhibitor.

N = 227 (%)	Ineligible for platinum with ICI (n = 131)	Eligible for platinum with ICI (n = 96)	p-value
Age (median), years	66	66	0.263
Sex			
Male Female	58 (44%) 73 (56%)	54 (56%) 42 (44%)	0.075
Smoking Status			
Never Former Active	1 (1%) 41 (31%) 89 (68%)	3 (3%) 34 (35%) 59 (62%)	0.302
Smoking years (median)	50	40	0.089
ECOG PS			
0-1 2 3-4 Unknown	6 (4%) 65 (50%) 56 (43%) 4 (3%)	96 (100%)	<0.001
CNS Metastases	57 (44%)	45 (47%)	0.615

N, number; ICI, immune checkpoint inhibitor; ECOG PS, Eastern Cooperative Oncology Group Performance Status, CNS, central nervous system

**Figure 3:**
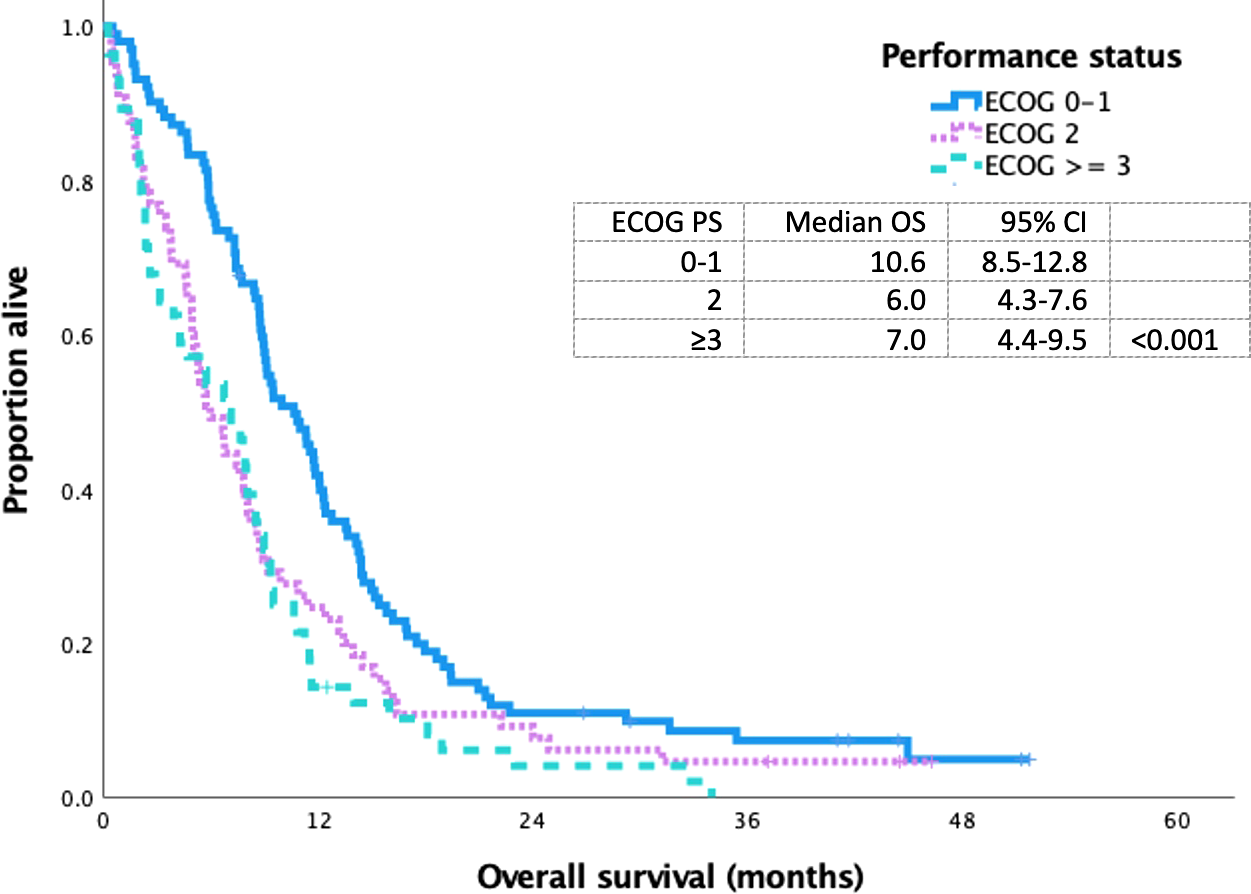
Kaplan Meier curve of extensive stage small cell lung cancer patients treated with platinum doublet chemotherapy based on ECOG 0-1 VS 2 VS ≥ 3.

The median OS for ES SCLC who received first-line platinum-doublet chemotherapy, non-platinum-doublet chemotherapy, and best supportive care was 8.4 (95% CI 7.6–9.3), 1.9 (95% CI 0.9–2.9), and 1.5 (95% CI 1.2–1.9) months (p<0.001), respectively. The median OS for PS 0–1, 2, and ≥3 for patients who received platinum doublet was 10.6 (95% CI 8.5–12.8), 6.0 (95% CI 4.3–7.6), and 7.0 (95% CI 4.4–9.5) months (p<0.001), respectively (**Figure 3**).

## Discussion

Our real-world SCLC population demonstrates that a minority of patients meet the clinical trial eligibility criteria for platinum doublet plus ICIs once this is publicly reimbursed in Canada. While a small subset of patients was excluded due to immunotherapy contraindications, the majority of the patients were not eligible on the basis of poor PS. Consequently, the evidence from the large phase III clinical trials, CASPIAN, and IMpower133, needs to be interpreted cautiously due to the extrapolation of the benefits to the real-world symptomatic ES SCLC patient.

Over half of our real-world ES PS population had an ECOG PS of ≥2 at diagnosis. While chemotherapy and ICI treatments have been widely used in good PS patients with lung cancer, due to the evidence in NSCLC, the feasibility is much less clear in PS 2 patients and is not currently informed by randomized clinical trials (16). A prospective randomized phase II clinical trial of ICIs with or without carboplatin and paclitaxel in advanced NSCLC with PS 2 is yet to be reported (17). In NSCLC consensus guidelines, expert opinion suggests that there are concerns regarding the safety and tolerability of combined chemotherapy plus single-agent immunotherapy with poor PS (18). Given the concerns regarding the toxicity of combination therapy with NSCLC, ES SCLC patients with PS ≥2 should be treated cautiously.

NSCLC and SCLC differ in the timing and rate of response to chemotherapy with the latter being more responsive in a shorter time frame (5). With this disease behavior, one may use combination therapy despite poor PS with the expectation that the disease would respond rapidly to chemotherapy. In our study, we examined the improvement in PS after one cycle of chemotherapy in patients with pretreatment PS ≥2 and noted that 17% improved to PS 0–1. As clinical improvement may take time, ECOG PS after two cycles was also collected and improved to PS 0–1 from PS ≥2 occurred in 18% of the patients who received at least three cycles. The significant attrition from cycle to cycle is notable; 17% of all platinum-treated patients only received one cycle, a reflection of the disease process and the tolerability of platinum doublet alone in this symptomatic population. A careful extension of the CASPIAN and IMpower133 data to selected PS ≥2 patients may be appropriate as treatment may result in a brisk improvement in the functional status (19).

An alternative strategy for poor PS patients may be a phased-in approach with platinum chemotherapy alone for the first cycle, followed by the addition of immunotherapy in the second cycle. It is unclear if similar survival benefits to the large phase III studies will be realized with this pragmatic approach. Similar to prior studies, in our cohort, poor PS remained a significant negative prognostic determinant, so it would be difficult to determine whether the difference in outcomes was a consequence of the PS or the alternate treatment scheme (7, 11). A stepped approach to treatment may mitigate toxicity risks and enable appropriate patients to receive ICIs with their platinum backbone.

Importantly, it must also be recognized that patients with poor PS (≥2) are not represented in CASPIAN or IMpower133; in addition, there is an important underrepresentation of older adults. This results in uncertainty of the clinical benefit in these patients (11). Additionally, the inclusion of patients with brain metastases in ES SCLC trials is important given their high prevalence (20). CASPIAN allowed the enrollment of patients with brain metastases; however, it required patients to be either asymptomatic or treated off steroids and anticonvulsants (12). IMpower133 required asymptomatic brain metastases to be treated prior to enrollment (14). This real-world study demonstrates that clinical trial eligibility criteria restrict enrollment and do not reflect the average patient with ES SCLC, compromising the external validity. This forces the clinician to practice with an evidence gap for patients who have a very narrow therapeutic window. While real-world evidence can act to supplement our knowledge, more pragmatic clinical trial design is needed for this symptomatic subset of lung cancer patients (18).

SCLC is a heterogenous malignancy with four subtypes defined by varied expression of transcription factors (21, 22). Conclusive biologic, molecular, and clinical markers have not been identified to help identify which ES SCLC will most benefit from ICIs; however, preliminary findings suggest that patients with an inflamed gene signature, based on transcription factors, may obtain the most benefit (21). One ongoing challenge with SCLC is the lack of targetable mutations due to the prevalence of tumor suppressor gene deletions and loss of function mutations as opposed to activating mutations (22). Currently, molecular profiling does not impact treatment selection, however it may in the future.

Our study is limited by the retrospective nature of this analysis. Past medical history and ECOG PS were collected through a manual chart review, which was limited by the accuracy of physician documentation. In addition, the other known prognostic factors for SCLC such as weight loss and laboratory values were not routinely collected. Our strengths include the real-world cohort representing a wide variety of baseline health states from a diverse geographic and socioeconomic population.

## Conclusion

Our real-world SCLC population demonstrates that by CASPIAN and IMpower 133 trial eligibility, up to 36% of patients who received platinum doublet would have been eligible for the addition of ICIs, 23% of the entire ES population. After one or two cycles of chemotherapy, an additional 11% of patients showed PS improvement to 0–1. While the results of the phase III studies are practice-changing, a significant proportion of ES patients do not meet the eligibility criteria. Clinical trials that are inclusive of poor PS patients will help address the evidence gap and will be practice-informing.

## Data availability statement

The datasets presented in this article are not readily available because of ethics approval. Requests to access the datasets should be directed to cho@bccancer.bc.ca.

## Ethics statement

This study was conducted with University of British Columbia/BC Cancer Research Ethics Board approval (H19-02381). A waiver of consent was granted to extract and analyze data for this retrospective review.

## Author contributions

RR was involved with data acquisition and data interpretation, drafted the original manuscript, and provided final approval of the manuscript. BL was involved with data acquisition and input into the original manuscript, and provided final approval of the manuscript. ZA-H was involved with data acquisition and input into the original manuscript, and provided final approval of the manuscript. CH was involved with research conception and design, data acquisition, and data interpretation, aided to the drafted original manuscript, and provided final approval of the manuscript version.

## Funding

This project has received research support from AstraZeneca. They did not have any involvement in the manuscript writing or interpretation of results.

## Conflict of interest

RR declared research grants from AstraZeneca. CH declared research grants from AstraZeneca, EMD Serono, and Roche. CH declared honoraria for advisory boards from AbbVie, Amgen, AstraZeneca, Bayer, BMS, Eisai, EMD Serono, Merck, Novartis, Pfizer, Roche, and Takeda.

The remaining authors declare that the research was conducted in the absence of any commercial or financial relationships that could be construed as a potential conflict of interest.

## Publisher’s note

All claims expressed in this article are solely those of the authors and do not necessarily represent those of their affiliated organizations, or those of the publisher, the editors and the reviewers. Any product that may be evaluated in this article, or claim that may be made by its manufacturer, is not guaranteed or endorsed by the publisher.
